# Psychological impact of COVID-19 containment on CADASIL patients

**DOI:** 10.1007/s00415-023-11648-8

**Published:** 2023-03-04

**Authors:** S. Reyes, A. Jabouley, N. Alili, M. H. De Sanctis, C. Machado, A. Taleb, D. Herve, N. Dias-Gastellier, H. Chabriat

**Affiliations:** 1 CNVT and Department of Neurology and Referral Center for Rare Vascular Diseases of the Brain and Retina (CERVCO), Hopital Universitaire Lariboisière, Assistance Publique des Hôpitaux de Paris, GHU-Paris-Nord, APHP, 2 Rue Ambroise Paré, 75010 Paris, France; 2 INSERM U1141–FHU-NeuroVasc, Paris, France

**Keywords:** COVID-19, Cerebral small vessel disease, CADASIL, Stroke, Depression, PTSD

## Abstract

**Introduction:**

COVID-19 restrictive containment was responsible for major psychological distress and alteration of quality of life (QoL) in the general population. Their impact in a group of patients having cerebral small vessel disease (SVD) and at high risk of stroke and disability was unknown.

**Objective:**

We aimed to determine the potential psychological impact of strict containment during the COVID-19 pandemic in a sample of CADASIL patients, a rare SVD caused by NOTCH3 gene mutations.

**Methods:**

Interviews of 135 CADASIL patients were obtained just after the end of the strict containment in France. Depression, QoL and negative subjective experience of the containment were analysed, as well as predictors of posttraumatic and stressor-related manifestations, defined as an Impact Event Scale-Revised score ≥ 24, using multivariable logistic analysis.

**Results:**

Only 9% of patients showed a depressive episode. A similar proportion had significant posttraumatic and stressor-related disorder manifestations independently associated only with socio-environment factors, rather than clinical ones: living alone outside a couple (OR 7.86 (1.87–38.32), unemployment (OR 4.73 (1.17–18.70)) and the presence of 2 or more children at home (OR 6.34 (1.35–38.34).

**Conclusion:**

Psychological impact of the containment was limited in CADASIL patients and did not appear related to the disease status. About 9% of patients presented with significant posttraumatic and stressor-related disorder manifestations which were predicted by living alone, unemployment, or exhaustion related to parental burden.

## Introduction

In France, at the heart of the COVID-19 epidemic in 2020 and well before the emergence of vaccination, major psychological distress and significant impairment of quality of life occurred in the general population at time of the containment [18, 27]. The most restrictive measures of containment occurred that year from March 17 and lasted 8 weeks. Accumulating data showed that populations under this lockdown could develop various mental disturbances, sleep difficulties, anxiety and posttraumatic and stressor-related disorder manifestations [41, 42, 44, 47]. Decompensation of premorbid psychiatric conditions and addictive behaviours were also frequent [6, 27]. In the Chinese population, 76% of individuals felt moderate-to-severe negative consequences using a self-report measure of posttraumatic subjective distress [41]. The analysis of such consequences was further complicated by the fact that the viral infection itself could also affect the brain tissue and lead to neuropsychiatric manifestations at individual level [19, 28, 34].

Predicting the negative impact of containment on the quality of life and psychological health was then crucial for developing preventive strategies at individual or familial level or specific policies at population level [6, 33, 42, 47]. Multiple predictors have been already identified in the general population, they were relating to age, sex, pre-existing psychological status, changes in the circadian rhythms, familial and social support, professional status, education level as well as cultural aspects [5, 20, 35, 36, 42]. Other factors are related to the living conditions such as the ability of teleworking, potential of home schooling, variety of occupational activities or exposure to daily information [14, 25, 28, 30, 33, 45].

At the end of the strict containment in France, we chose to investigate patients with a genetically-confirmed diagnosis of Cerebral Autosomal Dominant Arteriopathy with Subcortical Infarcts and Leukoencephalopathy (CADASIL). CADASIL is caused by mutations of the NOTCH3 gene that stereotypically lead to vascular accumulation of the derived protein products around smooth muscle cells and pericytes in the wall of cerebral microvessels [21, 22]. The disease is responsible for attacks migraine with aura, transient ischaemic attacks, repeated stroke and various psychological disturbances starting in midadulthood (mostly mood changes, anxiety and apathy). It leads progressively, after 50 to 60 years, to severe motor and cognitive decline [11].

At the beginning of the COVID-19 pandemic, CADASIL patients were informed that they were particularly exposed to an increased risk of stroke [40, 43, 46] since the viral infection could promote severe vascular inflammation and thrombosis [13]. We then hypothesized that CADASIL patients, who are already prone to anxiety and mood disturbances [8], might be particularly distressed during the very strict quarantine imposed at the onset of the COVID pandemic. Thus, we undertook a detailed survey for evaluating the psychological status and quality of life of these patients just at the end of the strict containment, together with their clinical status, social situation and daily living conditions during this time-frame.

The present study aimed at estimating the importance of distress in CADASIL patients during this exceptional event and to delineate potential predictors.

## Methods

### Subjects

Patients were selected based on the following inclusion criteria: (1) age higher than 18 years, (2) diagnosis confirmed by a genetic test and showing a typical cysteine mutation of the NOTCH3 gene, (3) participation to the French Cohort of CADASIL patients evaluated in the National Referral Centre for rare cerebrovascular diseases (www.cervco.fr); (4) having at least two previous follow-up visits in the centre, (5) lack of sensory or severe cognitive difficulties (particularly in reasoning, language or attention) that could compromise the understanding of questions, (6) French language skills allowing easy understanding of questions asked by phone, (7) informed consent already obtained for collecting clinical and imaging data during follow-up. Patients already known having dementia (defined by DSM-IV-TR criteria [3]), severe motor disability and who were highly dependent in daily life were excluded from the outset.

### Evaluation procedure

An experienced physician (NA) assessed all potential candidates who were pre-selected from the cohort on a first telephone interview. On this occasion, clinical information were updated for each subject, particularly any recent history of migraine with aura, stroke, mood disturbances and cognitive difficulties as well as the severity of disability based on the modified Rankin Scale (mRS) using a structured questionnaire [15]. Ongoing treatments including psychotropic medication were finally noticed. Thereafter, the physician checked whether the patient was willing and consent to be contacted by telephone and interviewed by a psychologist of the CERVCO team. The evaluation was then performed by experienced psychologists (SR, AJ, MH, CM) based on individual telephone interviews of 1–2 h, no later than 6 weeks after the end of the strict containment that occurred in France between 17th march and 10th may 2020.

### Information collected

A specific questionnaire dedicated to the pandemic situation was specifically developed based on the literature available at that time and considering the specificities of the genetic disease. The different questions and items were established by consensus among all the authors of the present report who had a long-term experience in the care of CADASIL patients (Table1). This questionnaire provided 3 different scores reflecting key issues regarding the containment: score 1—the level of stressors the patient was confronted with, score 2—the level of risk related to viral contamination and clinical worsening during the containment, score 3—the level of her/his subjective experience of the containment considered as negative. The score related to the level of stressors was varying between 0 (low) and 10 (high). Both the material conditions, isolation and stressing events were assessed, particularly, isolation or overcrowding in the patient’s residence, teleworking at home with or without the need of organising children's schooling, possibility of outdoor access (terrace or garden), difficulties in the economic situation including loss of employment, lack of contact with loved ones, occurrence of a severe form of COVID19 infection or death in close relatives of friends, difficulties in accessing health services. The second score related to the risk of worsening of health status associated to potential viral infection was varying from 0 (low) to 3 (high). This score was based on the evaluation of the COVID19 virus circulation around the patients and occurrence of infection in the patient. Finally, the third score related to the level of negative experience of containment could vary from 0 to 7, 7 corresponding to the worst experience of isolation the patient has ever had. This score was estimated based on the global feeling of isolation, perception that the cerebrovascular disease worsened this isolation, perceived anxiety related to daily news received from the media, difficulties in establishing daily routines, lack of motivation in everyday life and feeling that life will never be the same as before.

**Table 1:** Questionnaire developed by the CERVCO team and used by the psychologists for interviewing the CADASIL patients

Scoring	0	1
**Level of stressors previously identified in the general population related to the negative impact of containment**		
Were you living alone during the containment?	No	Yes
How many persons per room were you during the containment?	< 3	≥ 3
Did you have access to an open space (garden or balcony) during the containment?	Yes	No
Did you do teleworking during the containment?	No	Yes
Did you do teleworking while taking care of your children's schooling during the containment?	No	Yes
Did you loose your job due to the COVID-19 outbreak?	No	Yes
Did your socio-economic situation get worse because of the lockdown?	No	Yes
Did you hear from your loved ones during the containment?	Yes	No
Have any of your loved ones died or been in intensive care due to COVID-19?	No	Yes
Since the start of the epidemic, did you have difficulties to access medical care?	No	Yes
**Level of contamination in the patient's environment during the containment**		
Did you have numerous COVID-19 cases in your regional area during the containment?	No	Yes
Has anyone close to you been tested positive for COVID-19 during the containment?	No	Yes
Were you personally tested positive for COVID-19 during the containment?	No	Yes
**Level of negative subjective experience during the containment**		
Did you feel or perceive any positive aspect (s) during the containment ?	Yes	No
During the containment, did you consider your isolation as particularly difficult ?	No	Yes
Do you think that your cerebrovascular illness made this containment’s experience worse ?	No	yes
Do you think that media information was particularly anxiety-inducing during the containment?	No	yes
Did the containment completely disrupt your daily routines?	No	yes
During confinement, did you feel that your motivations were still intact?	Yes	No
During the containment, did you feel that your life will never be the same again?	No	yes

In addition to the questionnaire, quality of life was evaluated using the Euro-Qol [32], a standardised self-rated measure of quality of life related to the health status. We used the global measure of health status which was rated on a vertical visual analogue scale from 0, corresponding to the “worst imaginable health state”, to 100% corresponding to the ‘best imaginable health state’. We also used the anxiety/depression dimension having 3 levels of perceived difficulties (1: no problem, 2: some problems and 3: extreme problems). The French version of the Montgomery and Asberg Depression Rating Scale (MADRS) was used as a hetero scale assessing intensity of depressive symptoms from 0 (low) to 60 (severe) [12, 29]. The presence of a major depressive disorder was confirmed based on both a structured psychological interview assessing the corresponding DSM V depression criteria [1] and a MADRS score higher than 8, corresponding to mild symptoms [24, 38]. To asses traumatic and stressor-related symptomatology, psychologists applied by telephone the French version of the Weiss Impact of Event Scale (IES-Revised) [7, 42], a 22-item self-report measure widely used to assess multiple symptoms and particularly subjective distress caused by traumatic or stressor events. The IES-R yields a total score (ranging from 0 to 88) including 3 subscale scores for intrusion, avoidance, and hyperarousal, the core symptoms of posttraumatic stress disorder (PTSD). In the present study, a cut-off score equal or higher than 24 was considered as significant posttraumatic or stressor manifestations and a score higher than 33 as compatible with the diagnosis of PTSD [9].

Data obtained before the containment regarding the psychological status of all voluntary patients were also extracted from the database, the educational level and particularly the history of depressive episodes, degree of anxiety, global cognitive status, and quality of life at the last clinical follow-up preceding the containment. Additional data that may influence the effects of containment at individual level such as the marital status (living in couple or not), general socio-demographic characteristics (active life, retirement or unemployment) and number of children at home during the containment were also recorded.

### Statistical analysis

Data were first described using their percentage, mean, standard deviation, median, first and third quartile values. Normality was assessed with graphic analysis and the normal quantile plot and tested using the Shapiro Wilk test when needed.

Univariate analysis was then performed for comparison of socio-demographic and clinical data before containment and of severity of disability, depression and quality of life at time of the survey, between patients with IES-R score higher or equal to 24 and the others. The comparison was performed based on the Exact Fisher’s test for categorical variables, the Student’s *t* test for continuous variables of normal distribution and the Wilcoxon test in the absence of normality. Paired samples Wilcoxon test (signed rank test) was used to assess whether a significant modification of the modified Rankin Scale, the Anxiety/Depression levels of EQ5, the occurrence of a major depression or the quality of life changed between the last follow-up visit and the results obtained at time of containment.

Multivariable binomial logistic models were fit to find the final and best model using the Akaîke criterion to predict the presence of significant posttraumatic stress manifestations (IES-R ≥ 24) after selection of factors among those with *p* value < 0.25 in the previous univariate analysis. Only variables with less than 5% of missing values were used in the analysis. The missing data, if any, were replaced using five replicate imputations based on the chained equation method for multiple imputation (package mice with R). The model was internally validated using bootstrapping. Statistical analysis was performed using JMP14.3 (SAS) and RStudio, 2022.12.0.

## Results

### Patients’ clinical status before containment

Among 358 patients included in the cohort, 202 patients were eligible based on our selection criteria. Among these individuals 6 were already deceased at time of the study, 5 had become demented, 45 cases could not be contacted by telephone and 11 refused to participate. One hundred and thirty-five patients of mean age 57 ± 12 years (SD) were included in this study. None of them had been infected with covid19 before the interview. The main characteristics of the population are detailed in Table 2. The median time interval between the last follow-up visit at the referral centre and the neuropsychological evaluation was 21 months. Two third of patients were women, the same proportion was living in couple, 40% had a high level of education, one third were already retired while 14% were unemployed, all had children. At clinical level, the mean MMSE score was 28 ± 2.5 but ranged from 11 to 30. Globally, patients were not severely disabled, the mRS score was 0 to 1 in 3 out of 4 patients, only 9 patients had a moderate or severe disability. Fifty one percent of patients did not previously suffer from a stroke event and one in five patients currently used an anxiolytic or an antidepressant drug. About 16% of selected individuals already experienced a major depressive episode.

**Table 2:** Sociodemographic and clinical features of the study individuals at the time of the survey

Age mean (SD), median(Q_25_–Q_75_)	57.9 (11.9), 58 (48–69)
Gender: women	65.1% (*n = *88)
Education level (number of years at school)	
Range (mediane,Q_25_–Q_75_); Mean (Sd)	12.2 (3.6). 4–22 (12–15)
High level of education (≥ 12 years)	40.2% (*n = *54)
**Marital status during confinement**	
Not in couple-alone	34.8% (*n = *47)
Living in couple	65.1% (*n = *88)
**Socioprofessional category**	
Active working	50.3% (*n = *68)
Unemployment	14.1% (*n = *19)
Retirement	35.5% (*n = *48)
**Number of children**	
None	0%
One	46.6% (*n = *63)
Two or more	53.3% (*n = *72)
**Previous stroke events**	
None	51.1% (*n = *69)
One	32.5% (*n = *44)
Two or more	16.3% (*n = *22)
History of migraine (all types)	42.9% (*n = *58)
**Modified Rankin Scale **(*n = *119)	
0–1	74.8% (*n = *89)
2	17.8% (*n = *21)
3–4	7.5% (*n = *9)
Anxiety/depression perception (Euro-Quol Dimension 5), levels 1, 2, 3	50%,43%,7%
Major depressive disorder	9% (*n = *28)
MADRS Score, Mean (Sd), MEDIANE (Q_25_–Q_75_)	11.0 (9.9), 8 (2–18)
Current use of psychoactive drugs, % (*N*)	21.4% (*n = *29)
Antidepressant % (*N*)	17.7% (*n = *24)
Anxiolytic, % (*N*)	2.9% (*n = *4)
Antipsychotics, % (*N*)	0.7% (*n = *1)
Quality of life( EuroQuol Analogue Visual Scale) MEAN (SD), MEDIANE (Q_25_–Q_75_)	70.5 (18.8), 70 (60–85)

### Negative subjective experience during the containment in the whole sample

**Fig. 1:**
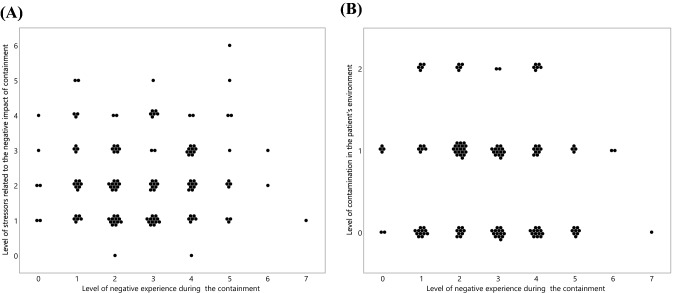
Distribution of cases showing the level of negative experience during the containment according to the level of stressors (**A**) or to the level of viral contamination (**B**)

Based on our dedicated questionnaire, only 15 patients had a level higher than 5 of negative subjective experience during the containment (score 3). A fraction of 15 to 20% of the sample had a level of 1, a similar fraction had a level 2, 3 and 4. Less than 5% did not experience any negative feeling. The level of this negative subjective experience was not related to the number of stressors presumably related to the negative impact of containment in the general population [(spearman rank (*ρ* = − 0.0454, *p = *0.6013)] nor to the level of contamination in the patient’s environment [(*ρ* = − 0.0998, *p = *0.247)] (Fig. 1).

### Psychological manifestations just after the containment in the whole sample

At the time of the survey, only 9% of patients (*n = *12) were suffering from a major depressive episode. Two among the latter, who did not previously experience depression, were using an antidepressant treatment. Only three already experienced depression but were not treated. Of the 21 patients who were depressed at the last visit before the interview, only 6 continued to use an antidepressant, 18 were no longer affected during the containment period.

In the whole population, 21% of patients (*n = *28) were currently using psychoactive drugs. When they were treated by an antidepressant drug, more than 90% of patients were using a selective serotonin reuptake inhibitor (SSRIs) (paroxetine, escitalopram, fluoxetine or sertraline). Only 3% of individuals were daily using anxiolytics (benzodiazepine drugs) and only one patient was treated by an antipsychotic drug (Table1).

Although depression was less present in the whole sample after the containment then before (difference of means (*δ*) = 0.08, *p = *0.02), a small but significant increase of the mRS score (δ: 0.20, *p < *0.02) and of the MADRS score (*δ = *2.6, *p = *0.0033) was detected at time of the survey. Conversely, the variation between pre and post-containment of the Euroquol global visual score and of the Euroquol quantitative score evaluation of anxiety/depression dimension did not vary significantly in the whole sample (*δ = *0.02, *p = *0.9 and *δ = *0.07, *p = *0.22 respectively).

### Frequency and predictors of posttraumatic or stressor-related manifestations (partial posttraumatic disorder and probable posttraumatic disorder)

One patient did not provide all answers to the IES-R questionnaire, in the other 134 patients, the mean IES-R was 9.4 ± 10.7 (median: 5, Q1–Q4: 2–14). Only 13 patients, had an IES-R score larger than or equal to 24. Among them, 7 had a score equal or higher than 33. These two groups were compared to patients with IES-R less than 24 (Table 3). Patients with IES-R score ≥ 24 had a higher Anxiety/Depression EQ5 level, were 5 times more often depressed and had a higher MADRS score than patients with IES-R score less than 24. In contrast, the mRS and Visual Euroqol scores did not differ between these groups. The different IES-R subscores were all increased without any distinction for one of the 3 subscores. Similar contrasts were observed when the analysis was restricted to patients with IES-R score ≥ 33.

**Table 3:** Impact of Event Scale Revised: contrasting features between patients with score < 24, ≥ 24 and ≥ 33

IES-R global score limits	< 24 (*n = *121)	≥ 24 (*n = *13)	*P* value	≥ 33 (*n = *7)	*p* value^a^
**Sociodemographic aspects**					
Age	58.04 ± 11.95	55.23 ± 11.07	*0.389*	53.8 ± 10.83	*0.33*
Female sex	**62%**	**100%**	***0.003***	**100%**	***0.04***
Education years > 12	41%	31%	*0.563*	43%	*1*
Living alone vs in couple	**32%**	**62%**	***0.036***	**71%**	***0.04***
**Professional status**					
Active working	**53%**	**31%**	***0.012***	**43%**	***0.09***
Unemployment	**11%**	**43%**		**43%**	
Retirement	**36%**	**26%**		**14%**	
Two children or more	52%	77%	*0.088*	57%	*1*
**Clinical data available before containment**					
MMSE score	28.1 ± 2.6	27.5 ± 2.3	*0.31*	28.3 ± 1.97	*0.81*
Previous stroke events	49%	46%	*1*	42%	*0.74*
Past history of migraine	43%	39%	*0.776*	14%	*0.24*
mRS (2–5 vs 0–1)	**21%**	**53%**	***0.021***	40%	*0.32*
Anxiety/Depression (EQ 5) Levels 1, 2, 3	46–46–8%	23–61–15%	*0.252*	43–43–28%	*0.19*
Major depressive disorder	16%	23%	*0.450*	14%	*1*
MADRS score	**7.76 ± 7.23**	**12.5 ± 2.12**	***0.07***	11.72	*0.26*
Quality of life(EuroQuol Analogue Visual Scale)	71 ± 18	62.7 ± 22.9	*0.21*	69 ± 27	*0.84*
**Information at time of containment**					
mRS (2–5 vs 0–1)	34%	46%	*0.54*	28%	*0.8*
Anxiety/Depression (EQ Dimension 5) Levels 1, 2, 3	**54–43–3%**	**15–46–38%**	***0.0003***	**14–14–43%**	** < *0.0001***
Major depressive disorder	**6%**	**31%**	***0.01***	**43%**	***0.01***
MADRS	**9.98 ± 9.69**	**20.40 ± 7.79**	***0.003***	**21.9 ± 7.9**	***0.003***
Quality of life( EuroQuol Analogue Visual Scale)	70.42 ± 19.28	62.0 ± 5.58	*0.29*	60.28 ± 29.42	*0.41*
Level of stressors > 4	15%	23%	*0.42*	14%	*1*
Level of contamination	56%	61%	*0.77*	71%	*0.69*
Level of negative experience > 5	9%	23%	*0.17*	14%	*0.53*
IES-R subscore intrusion	**2.6 ± 2.8**	**12.9 ± 4.4**	** < *0.0001***	**14.1 ± 5.3**	** < *0.0001***
IES-R subscore avoidance	**2.0 ± 2.8**	**11.2 ± 6.3**	** < *0.0001***	**13.6 ± 6.9**	** < *0.0001***
IES-R subscore hyperarousal	**2.1 ± 2.6**	**10.6 ± 5.4**	** < *0.0001***	**13.5 ± 4.7**	** < *0.0001***

Significant differences are in bold

^a^Comparison with the reference group IES-R, Wicoxon Rank tests were used for continuous values, Fischer’s Exact test for %

Multivariable analysis showed that the best predicting model of IES-R ≥ 24 in patients (ROC curve AUC = 0.81) included 4 factors, 3 of which were predictors of significant post-stress manifestations: (1) the status of unemployment (OR = 4.73, *p < *0.03), (2) the status of being single and not in couple (OR = 7.86, *p = *0.0047) and 3) the fact to have more than one child (OR = 6.34, *p = *0.018) at home during the containment (Table 4). The different clinical parameters that differ in univariate analysis according to the level of IES-R did not improve the prediction model. No interaction was detected between the factors identified. Internal model validation showed a Somers D value of 0.643, c index of 0.822 (proportion of patients ordered correctly by the model) and R^2^ of 0.373.

**Table 4:** Results of multivariate regression analysis (3 significant predictors–AUC = 0.821)

Term	Estimate	SE	Chi2	*p* Chi2	Lower 95%	Upper 95%	Odds Ratio	*p*	Lower 95%	Upper 95%
Intercept	− 3.69	0.95	15.01	0.0001*	− 5.84	− 2.05	–	–	–	
Unemployment vs work/retirement	− 0.78	0.35	5.01	0.0252*	− 1.46	**− 0.08**	**4.73**	0.0295*	**1.17**	**18.70**
Alone vs Couple	1.03	0.38	7.39	0.0066*	0.31	**1.82**	**7.86**	0.0047*	**1.87**	**38.32**
More than 1 child	1.85	0.83	4.90	0.0269*	0.30	**3.65**	**6.34**	0.0180*	**1.35**	**38.34**
Anxiety/Depression (EQ 5) Levels 1 vs 2/ 3	1.09	0.76	2.05	0.15	− 0.31	2.75	2.95	NS	0.73	15.71

Significant differences are in bold

## Discussion

The results of this study showed that the strict containment ordained in France over 8 weeks in 2020 to reduce the COVID 19 contamination had, globally, a limited impact on mental health in a sample of CADASIL patients. Just at end of the containment, only 9% of individuals were found depressed whereas this frequency was about 16% at their last follow-up visit. More specifically, 18 patients who were previously depressed were no longer affected at time of the containment. This low prevalence contrasts with the 16.5% frequency of depression estimated in a survey obtained in the general population at the initial outbreak among 1738 respondents from 190 Chinese cities [42]. It was also lower than the 17% prevalence of depression estimated in the general population using an internet-based-questionnaire in Italy just after the first lockdown measures [35]. Differences in the definition of depression, type of survey and selection biases among responders might well explain this discrepancy. It should also be noted that 21% of the cohort individuals were already treated by an antidepressant or an anxiolytic drug at time of containment which could have significantly mitigated the impact of the lockdown on mood of CADASIL patients. In line, only a small increase of the MADRS score was detected in the whole cohort at end of the containment, without any significant impact on the quality of life. The degree of negative experience felt related to this exceptional event was assessed at individual level. A score higher than 5 was obtained only in 15 patients based on 7 specific questions related to the lack of positive output, degree of isolation, risk for health, anxiogenic daily news, routine disruption, demotivation and global life-altering effect in relation to the containment [17, 33, 41, 47]. This discomfort did not appear related to the number of stressors nor to the risk level of viral contamination in the CADASIL patient's environment. Altogether, these data support that the immediate effects of containment were actually limited in this sample of patients, that there was no effect on the frequency of major depressive episodes, and some psychological impact limited to a subgroup of individuals.

Exposure to an exceptional event as the “lockdown” could also have precipitated transient or more enduring psychological manifestations related to posttraumatic stress or stressor-related disorder independently from depression [16]. The mean IES-R score of 9.4 measured in our CADASIL patients was 3 times lower than that reported in a much larger number of volunteers at time of the outbreak in China [42]. Our result was much closer to the mean IES-R value measured among non-medical health care workers during the COVID crisis in Singapore and which was twice higher than that detected in the medical staff in the midst of the COVID 19 outbreak [39]. In the present study, 13 out of 135 CADASIL patients had an IES-R score equal or higher than 24, a level considered as best predicting the occurrence of significant manifestations of a posttraumatic stress disorder [4]. Among them, 7 reached a score higher than 33, a cut-off that provides a good accuracy level for PTSD [9]. They also showed, as expected, that CADASIL individuals with significant posttraumatic or stressor-related manifestations had a higher MADRS score and were 5 times more depressed than the other patients.[9]. In this subgroup of patients, the reduction of quality of life was limited and did not reach statistical significance as also the increase level of negative experience. The parallel increase of the 3 IES-R subscores suggest that the development of posttraumatic or stress manifestations does not follow a specific pattern but involved both hyperarousal physical symptoms, avoidance behaviours to contain the distress and intrusive distressing memories, in a similar way.

CADASIL patients with posttraumatic or stressor-related manifestations were found more frequently to be women, living alone, unemployed, and having more neurological disability in univariate analysis. However, only 3 socio-environmental factors predicted independently the occurrence of posttraumatic or stressor-related manifestations. All these factors had been already recognized as potentially capable to precipitate the occurrence of stress manifestations during the COVID 19 pandemic in the general population [17, 33, 41, 47]. First, CADASIL patients who were living alone were found to present posttraumatic or stressor-related symptoms nearly eight times more frequently than patients who were living in a couple. Outside this context of COVID pandemic, singlehood has been already shown to have a considerable impact on the psychological health of individuals in the general population [2]. In individuals living alone, the lockdown situation obviously aggravated this isolation by reducing or completely eliminating the interactions which were previously maintained through working activities [31, 37]. The absence of a close partner with whom the patient can share his/her impressions, routines of daily life, hobbies and can discuss the information received through the media appears to be particularly deleterious. Second, the results showed that CADASIL patients who were unemployed were 4.7 times more likely to develop posttraumatic or stressor-related symptoms than the rest of the sample. Clearly, unemployment had probably further aggravated the social isolation during the lockdown and was already shown to be a major concern during the COVID pandemic in a previous UK-based focus group study [44]. The unemployed situation increases the uncertainty felt by the patient about his/her social reintegration and his/her future. It can also aggravate or decompensate an already precarious economic situation, another source of psychological stress [26]. Third, patients who had two children and more at home were about 6 times more likely to develop posttraumatic or stressor-related manifestations than the rest of the population. This could be related to the difficult management of organising courses and working at home in parallel, to the inherent communication problems depending on the quality of the technical installation, access to internet, and schedules, but also to the difficulty in maintaining the attention of children for hours of work for lessons at home in these circumstances [10, 23]. The premises, the number of people per room, the noise generated by the various activities, are all potential additional sources of complications.

We are fully aware of different weaknesses of the present study. The selection of our patients was necessarily biased because we chose to investigate only individuals from a cohort of volunteers, who already accepted to be followed and for whom information could be easily collected. Thus, this sample could not represent the whole population of CADASIL patients. Moreover, the study population was relatively small in size which prevented the identification of other predictors of post-containment manifestations. We also did not validate externally the present results in an independent population. Thus, it is most likely that the present findings might not be transposed to a different CADASIL cohort in a distinct social, cultural or political context. For instance, the lack of predictors related to the clinical or psychological status observed in the present study might be specifically related to the situation of individuals who were diagnosed in France, who could benefit from a 100% coverage of all their health care costs as well as a full wage replacement throughout the entire period of containment. The present study also had a number of strengths. All investigations were carried out in a homogeneous population corresponding to patients at the highest risk of decompensation who already had a confirmed genetic diagnosis of their disease and without major disability. This study was also based on clinical tools and scores already validated. Detailed clinical information were available for each individual. Clinical and psychological information were updated and collected through direct intervention of professionals with a long expertise in the management of CADASIL. The results were also coherent. They showed without any ambiguity the importance of socio-environmental factors that could worsen the psychological status of CADASIL patients during the lockdown period.

## Conclusion

In conclusion, we believe that the results of this study support that isolation of CADASIL patients, unemployment and the number of children at home should be considered to evaluate the risk of psychological distress during exceptional situations leading to social breakdown as the containment.

## Data Availability

Data related to this study are available upon request.
